# Supplementary material for: Identification of Veillonella parvula and Streptococcus gordonii adhesins mediating co-aggregation and its impact on physiology and mixed biofilm structure
Source: mBio. 2024 Nov 11;15(12):e02171-24. doi: 10.1128/mbio.02171-24 (PMC11633186; doi:10.1128/mbio.02171-24)
Supplement: Supplemental material — Tables S1 and S2; description and link for Data S1 to S3; Figures S1 to S12; supplemental methods. [file mbio.02171-24-s0001.pdf]

Supplementary Material

Supplementary Tables

Supplementary Table S1 : list of strains used in this study

Strain	Description	Reference
Veillonella parvula **SKV38 strains**		
SKV38	WT	(Knapp et al. 2017)
ΔvmaA mutant	SKV38 *ΔFNLLGLLA_00032*::*tetM*	(Béchon et al. 2020)
ΔvmaB mutant	SKV38 *ΔFNLLGLLA_00335*::*tetM*	(Béchon et al. 2020)
ΔvmaC mutant	SKV38 *ΔFNLLGLLA_00581*::*tetM*	(Béchon et al. 2020)
Δ*vtaA* mutant	SKV38 *ΔFNLLGLLA_00516*::*tetM*	(Béchon et al. 2020)
ΔvtaB mutant	SKV38 *ΔFNLLGLLA_00034*::*tetM*	(Béchon et al. 2020)
ΔvtaC mutant	SKV38 *ΔFNLLGLLA_00038*::*tetM*	This study
ΔvtaD mutant	SKV38 *ΔFNLLGLLA_00044*::*tetM*	This study
ΔvtaE mutant	SKV38 *ΔFNLLGLLA_00045*::*tetM*	This study
ΔvtaF mutant	SKV38 *ΔFNLLGLLA_00046*::*tetM*	This study
ΔvtaG mutant	SKV38 *ΔFNLLGLLA_00098*::*tetM*	(Béchon et al. 2020)
ΔvtaH mutant	SKV38 *ΔFNLLGLLA_00099*::*tetM*	(Béchon et al. 2020)
ΔvtaI mutant	SKV38 *ΔFNLLGLLA_01790*::*tetM*	(Béchon et al. 2020)
ΔvtaA ΔvtaC-F mutant	SKV38 *ΔFNLLGLLA_ 00516*::*catP* *ΔFNLLGLLA_00036-46*::*tetM*	(Béchon et al. 2020)
ΔvtaC-F mutant	SKV38 *ΔFNLLGLLA_00036-46*::*tetM*	(Béchon et al. 2020)
P_{Tet} -*vtaA* mutant	SKV38 *catP-Term(fdx)*-P*tet-FNLLGLLA_00516*	(Béchon et al. 2020)
P_{Tet} -*vtaE* mutant	SKV38 *ermE-Term(fdx)*-P*tet-FNLLGLLA_00045*	This study
Δ*vtaE* Δ*vtaC* mutant	SKV38 *ΔFNLLGLLA_ 00045*::kanR *ΔFNLLGLLA_00043*::*tetM*	This study
Δ*vtaD-E* mutant	SKV38 *ΔFNLLGLLA_00044-45*::*tetM*	This study
Δ*vtaE* Δ*vtaF* mutant	SKV38 *ΔFNLLGLLA_ 00045*::kanR *ΔFNLLGLLA_00046*::*tetM*	This study
Δ*vtaD-E* Δ*vtaA*	SKV38 *ΔFNLLGLLA_00044-45*::*tetM* ΔFNLLGLLA_ 00516::catP	This study
Streptococcus gordonii **DL1**		
DL1	WT	(Nairn et al. 2020)
Δ*srtA*	DL1 Δ*SGO_1230*	(Nairn et al. 2020)
Δ*1487*	DL1 Δ*SGO_1487*	(Nairn et al. 2020)
Δ*abpB*	DL1 Δ*SGO_0162*	(Nairn et al. 2020)

Δ*cshB*	DL1 Δ*SGO_1148*	(Nairn et al. 2020)
Δ*hsa*	DL1 Δ*SGO_0966*	(Nairn et al. 2020)
Δ*sspAB*	DL1 Δ*SGO_0210-0211*	(Nairn et al. 2020)
Δ*0107*	DL1 Δ*SGO_0107*	(Nairn et al. 2020)
Δ*0208* /Δ*endoD*	DL1 Δ*SGO_0208*	(Nairn et al. 2020)
Δ*0316*	DL1 Δ*SGO_0316*	(Nairn et al. 2020)
Δ*0317*	DL1 Δ*SGO_0317*	(Nairn et al. 2020)
Δ*0385*	DL1 Δ*SGO_0385*	(Nairn et al. 2020)
Δ*0388*	DL1 Δ*SGO_0388*	(Nairn et al. 2020)
Δ*0405*/Δ*strH*	DL1 Δ*SGO_0405*	(Nairn et al. 2020)
Δ*0430*	DL1 Δ*SGO_0430*	(Nairn et al. 2020)
Δ*0707*	DL1 Δ*SGO_0707*	(Nairn et al. 2020)
Δ*0854*/Δ*cshA*	DL1 Δ*SGO_0854*	(Nairn et al. 2020)
Δ*0890*	DL1 Δ*SGO_0890*	(Nairn et al. 2020)
Δ*1182* / Δ*pavB*	DL1 Δ*SGO_1182*	(Nairn et al. 2020)
Δ*1247* / Δ*Nt5e*	DL1 Δ*SGO_1247*	(Nairn et al. 2020)
Δ*1415*	DL1 Δ*SGO_1415*	(Nairn et al. 2020)
Δ*1435*	DL1 Δ*SGO_1435*	(Nairn et al. 2020)
Δ*1486* / Δ*bgaA*	DL1 Δ*SGO_1486*	(Nairn et al. 2020)
Δ*abpA*	DL1 Δ*SGO_2105*	(Nairn et al. 2020)
Δ*1650*	DL1 Δ*SGO_1650*	(Nairn et al. 2020)
Δ*1651*	DL1 Δ*SGO_1651*	(Nairn et al. 2020)
Δ*1727*	DL1 Δ*SGO_1727*	(Nairn et al. 2020)
Δ*2004*	DL1 Δ*SGO_2004*	(Nairn et al. 2020)
Δ*padA*	DL1 Δ*SGO_2005*	(Nairn et al. 2020)
IDFC2-visA	DL1 IDFC2-*SGO_2004*	This study
P_{Tet}-visA	DL1 pTeT-pVeg(RBS)-*SGO_2004*	This study
Δ*padA* P_{Tet} -visA	DL1 Δ*padA* pTeT-pVeg(RBS)-*SGO_2004*	This study
***Escherichia coli* strains**		
DH5α	WT	
Bl21(DE3) pDIA17	WT	

Bl21(DE3) pDIA17 pET22b-*visA*-HIS	pET22b-*visA*-His	This study
**Other bacteria**		
S. oralis CIP103216	WT	CIP Institut Pasteur
S. parasanguinis CIP104372T	WT	CIP Institut Pasteur
S. gordonii CIP105038	WT	CIP Institut Pasteur
S. gordonii CIP105258T	WT	CIP Institut Pasteur
S. gordonii NCTC7868	WT	CIP Institut Pasteur
S. mitis CIP104996	WT	CIP Institut Pasteur
S. mitis CIP104997	WT	CIP Institut Pasteur
A. oris CIP102340	WT	CIP Institut Pasteur
F. nucleatum CIP101130T	WT	CIP Institut Pasteur
S. mutans NG8	WT	Justin Meritt(Lee et al. 1989)
S. mutans UA140	WT	(Switalski and Butcher 1994)
S. mutans UA159	WT	(Ajdić et al. 2002)8
S. mutans CBSm8	WT	Justin Meritt
S. mutans CBSm38	WT	Justin Meritt

Supplementary Table S2 : primers used in this study

name	Sequence (5'->3')	construction
0045-5F	actaaagatggcctcaac	Upstream of Ptet-*vtaE*/Δ*vtaE*-KanR
5R_pTet_VtaE	tcaacacactcttaagtttgcttcctcatcttactccttgcataaaac	Upstream of Ptet-*vtaE*
JW264	gaagcaaacttaagagtgtgttga	Ptet-ery cassette
K7ind-ptet-cat-R	aaaattttctcctttactgcagg	Ptet-ery cassette
3F_ptet_VtaE	cctgcagtaaaggagaaaattttatgaaccgaatttttaaagtaatctg	Downstream of Ptet-*vtaE*
0044_3R	ccgcctttagatgccttc	Downstream of Ptet-*vtaE*
5R_delta_vtaE_kana	cactcgcctgcattttactcatcttactccttgcataaaac	Upstream of Δ*vtaE*-KanR
F_kana	TAAAATGCAGGCGAGTGAAGaaaattttc	KanR cassette
R_kana	TAAAACAATTCATCCAGTAAAATATAATATTTTATTTTCTCCC	KanR cassette
3F_delta_vtaE_kana	ATTTTACTGGATGAATTGTTTTAGttcacatctgtttcatcttatattc	Downstream of Δ*vtaE*-KanR
0045_3R	gccaaggagttaaaaccc	Downstream of Δ*vtaE*-KanR

5F_SGO2004prom	gattccgaataaggctactg	Upstream of pTet-*SGO2004*/IDFC2-*SGO2004*
5R_SGO2004prom_IDFC	GATGAGCGTTATCGTGGCTCttaatgctttggttttcttctc	Upstream of IDFC2-*SGO2004*
F_IDFC_sgordonii	GAGCCACGATAACGCTCATC	IDFC cassette
R_IDFC_sgordonii	ttatttcctcccgttaaataatagataac	IDFC cassette
3F_2004prom_IDFC	GTTATCTATTATTTAACGGGAGGAAATAAatggatagaaaaaagttaattaagttag	Downstream of IDFC2-*SGO2004*
3R_2004_prom	cttggtaacgagcttcatac	Downstream of pTet-*SGO2004*/IDFC2-*SGO2004*
5R_SGO2004_pTet	gcagaattcgcccttaagtcttaatgctttggttttcttctc	Upstream of pTet-*SGO2004*
F_pTet_promoter	gacttaagggcgaattctgc	Ptet-pVEG_RBS cassette
R_pTet_pVEG_RBS	GTTTGTCCTCCTTATTAGTTAATCAagctcagatctgttaacgcta	Ptet-pVEG_RBS cassette
3F_2004_pTET_pvegRBS	TGATTAACTAATAAGGAGGACAAACatggatagaaaaaagttaattaagttag	Downstream of pTet-*SGO2004*
5F_delta_padA	gagcggatcagtatatcagtg	Upstream of Δ*padA* pTet-*SGO2004*
5R_delta_padA_pTetSGO	gcagaattcgcccttaagtcgctattttaaagcctatataaatagtag	Upstream of Δ*padA* pTet-*SGO2004*
F_SGO2004_pet22b	CTTTAAGAAGGAGATATACATatgcaaccagagattacgactcag	*SGO_2004*(aa136-698) for pET22b
R_SGO2004_pet22b	TCAGTGGTGGTGGTGGTGGTGttccttagtaccaatttcaactac	*SGO_2004*(aa136-698) for pET22b
pET22b_LINEAR_F	CACCACCACCACCACCACT	linearization of pET22b
pET22Bb_LINEAR_R	ATGTATATCTCCTTCTTAAAGTTAAACAAAATTATTTC	linearization of pET22b

Supplementary Data

Supplementary data S1 and S2 contain transcriptomic information (gene, annotation, Log2Fold, and P-value or list of significative comparisons) for both *V. parvula* and *S. gordonii*

Supplementary data S1 contains the data concerning *V. parvula* present on 6 different sheets:

- Genes_specific_coculture: contains all genes dysregulated in **all co-culture** conditions
- specific_aggregation_coculture: contains all genes dysregulated in all co-culture **with some type of aggregation** conditions
- genes_specific_noaggregation_c: contains all genes dysregulated in all co-culture **with no aggregation** conditions
- All_genes_with_important_logfol: contains all genes dysregulated in **at least one condition.**
- raw_data: Contains the raw RNA-seq data.
- gene_correspondance: correspondance to the genes and their different annotations.

Supplementary data S2 contains the data concerning *S. gordonii* present on 3 different sheets:

- Genes_specific_coculture: contains all genes dysregulated in **all co-culture** conditions
- All_genes_with_important_logfol: contains all genes dysregulated in **at least one condition.**
- raw_data: Contains the raw RNA-seq data.

Supplementary data S3 contains the parameters calculated for each image in BiofilmQ.

- Condition: pair of bacteria present in the image
- Experiment: replicate number (exp1, 2 or 3)
- unique_id_well : unique identifier of the image
- column and row: column and row localisation within the plate
- image number: corresponding to one of the four set position in each well.
- channel: BacGO or Syto61, corresponds to the imaging channel. All other columns contain the information of parameters calculated by BiofilmQ.See https://drescherlab.org/data/biofilmQ/docs/ for more information.

Supplementary data are available at https://github.com/ldorison/Coaggregation_streptococcus_Veillonella-

Supplementary figures

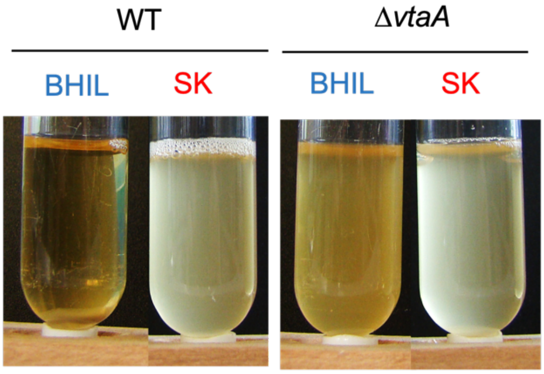

Supplementary Figure S1: *V. parvula* auto-aggregation phenotype in SK and BHI.
Overnight cultures of *V. parvula* WT and Δ*vtaA* in SK and BHIL medium.

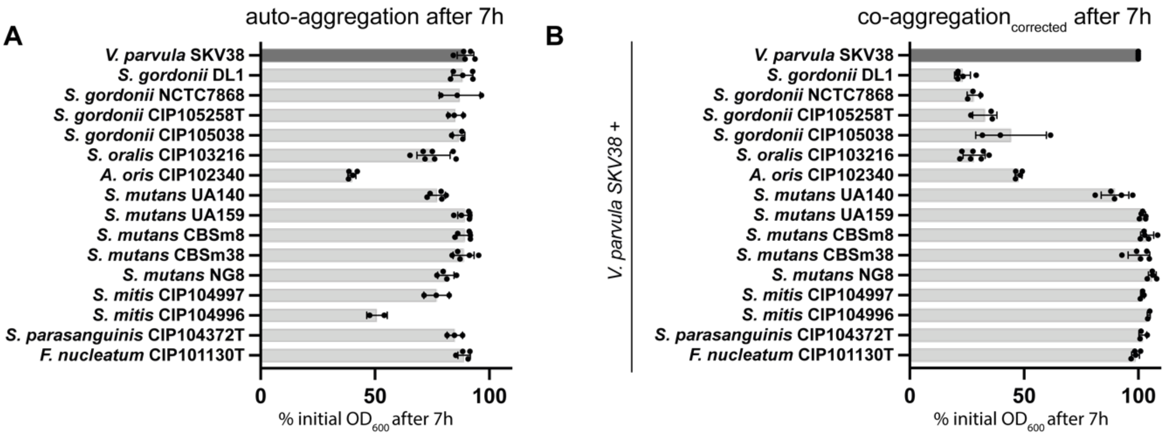

Supplementary Figure S2: Auto-aggregation and co-aggregation between *V. parvula* SKV38 and various members of the dental plaque.

(A) Auto-aggregation of *V. parvula* SKV38 and various members of the dental plaque after 7h, as measured by the % of decrease of optical density between 0 and 7h. 0 % of initial OD corresponds to full auto or co-aggregation while 100% of initial OD corresponds to no auto or co-aggregation. SD and single points for 3-5 replicates are shown. (B) Co-aggregation from independent cultures corrected for auto-aggregation of *V. parvula* SKV38 with various members of the dental plaque after 7h, as measured by the % of decrease of optical density between 0 and 7h. SD and single points for 3-5 replicates are shown. Correction was applied using the following formula:

$$Coaggregation_{corrected} = Coaggregation + 100 - \left(\frac{autoaggregation_{Vp} + autoaggregation_{partner}}{2}\right)$$

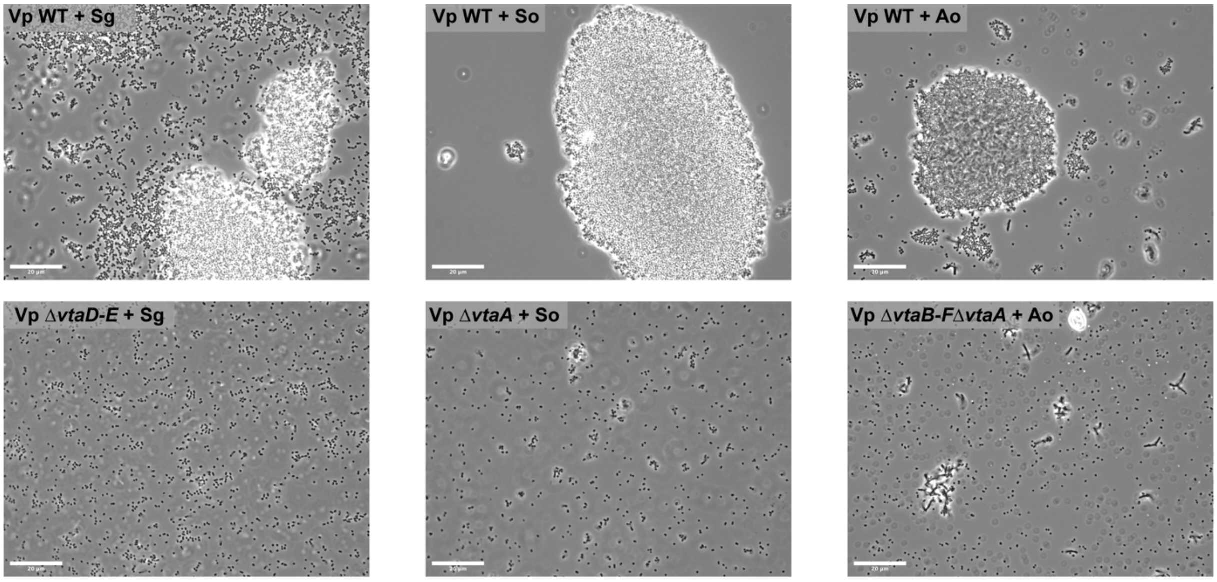

Supplementary Figure S3: Light microscopy images of co-aggregates between *V. parvula* SKV38 and *S. oralis*, *S. gordonii* and *A. oris*.

Brightfield images of aggregates after 7h of co-aggregation. 3 µL of bacteria suspension was sampled at the bottom of a cuvette used for an aggregation assay, mounted on a slide and observed under the microscope. Scale bar is 20 µm. Sg: *S. gordonii*, So: *S. oralis*, Ao: *A. oris*.

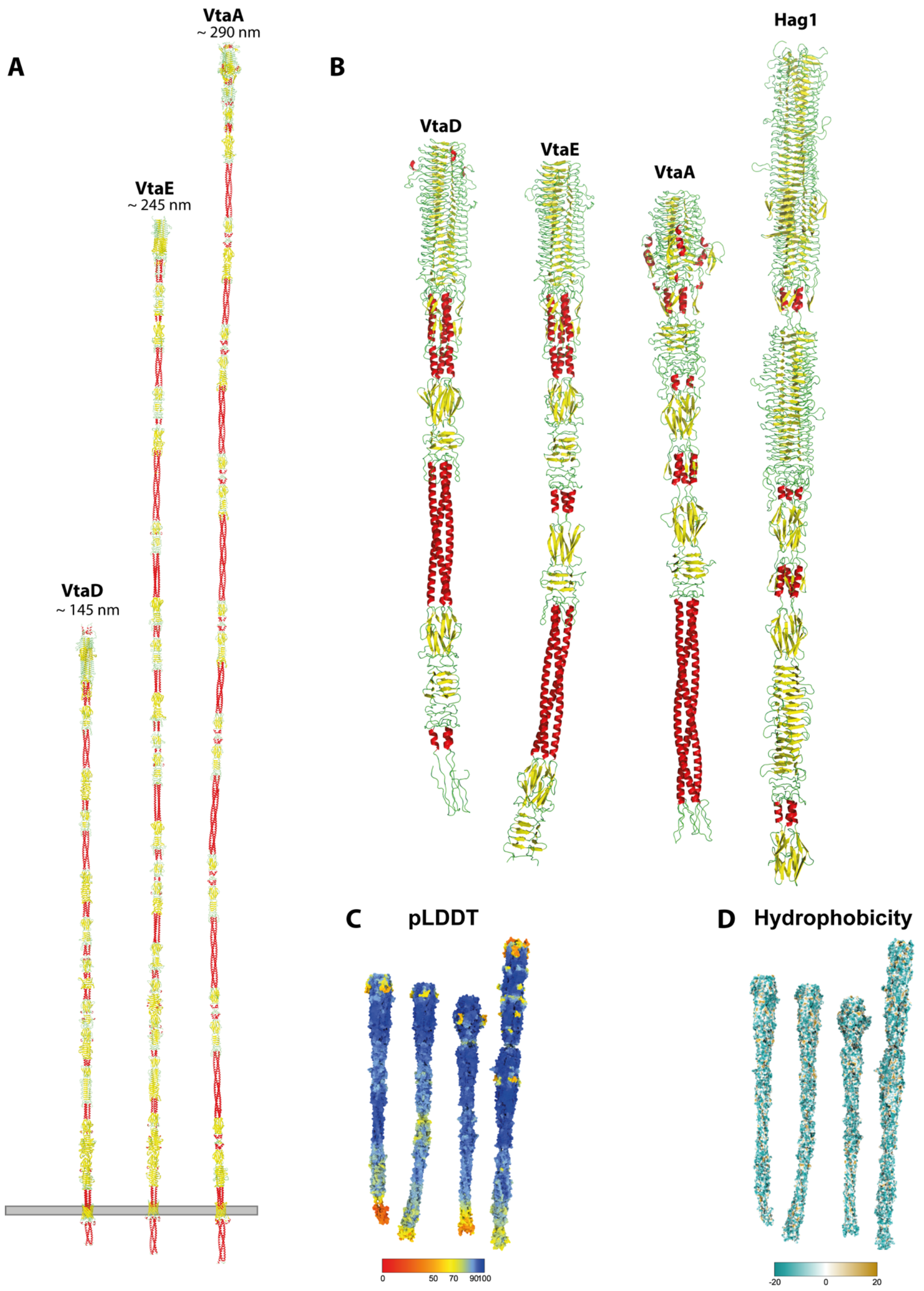

Supplementary Figure S4: Comparison of the predicted structures of *V. parvula* VtaD, VtaE, VtaA and *V. atypica* Hag1.

(A) Model predictions of the full length trimeric VtaD, VtaE and VtaA. Models were obtained by predicting 400-700 residues overlapping fragments of each adhesin as trimers using AlphaFold 2.3(Evans et al. 2022) locally. Fragments were then assembled in PyMOL using the command Super. (B-C-D) Comparison of the head structure, pLDDT (per-residue model confidence score, higher values mean higher confidence in the prediction) and hydrophobicity of VtaD, VtaE, VtaA and Hag1. Images, pLDDT coloring and hydrophobicity calculation were done in ChimeraX(Pettersen et al. 2021).

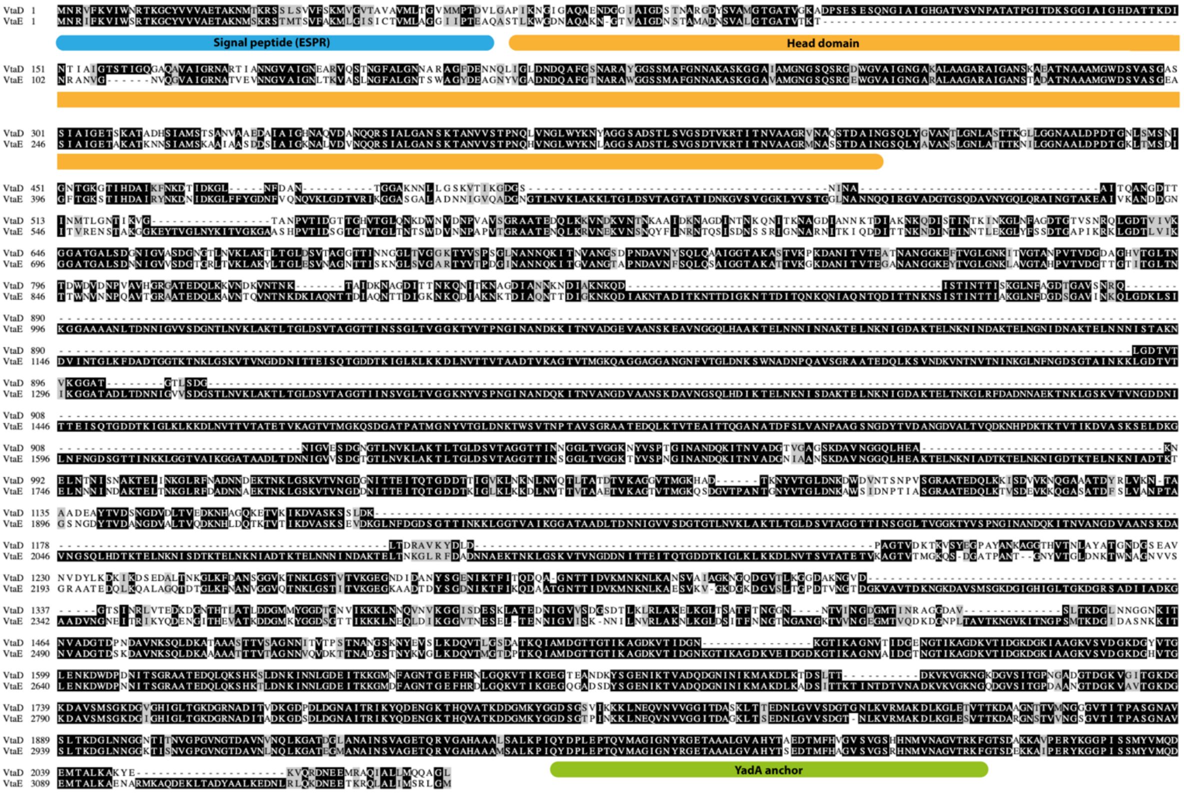

Supplementary Figure S5: Sequence alignment between *V. parvula* VtaE and VtaD.

Both protein sequences were aligned using the Emboss Water algorithm(Madeira et al. 2022). The ESPR sequence is highlighted in blue, the head section of the adhesins in orange and the YadA anchor in green. Identical amino acid sequences are highlighted in black, similar in gray and divergent in white.

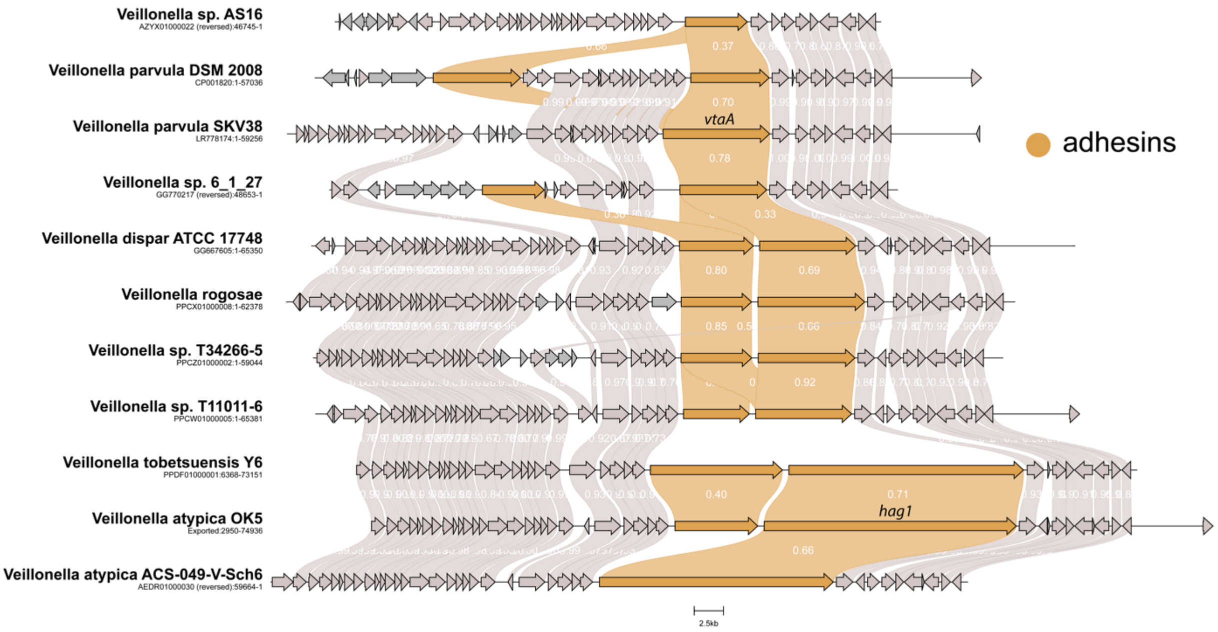

Supplementary Figure S6: Variations at the *vtaA* locus in *Veillonella*.

A local subset of *Veillonella* genomes was searched for presence of the *vtaA* neighboring MATE efflux transporting using the HMM profile PF01554.21. Neighboring regions were extracted and aligned using clinker(Gilchrist and Chooi 2021). Trimeric autotransporters are colored in brown.

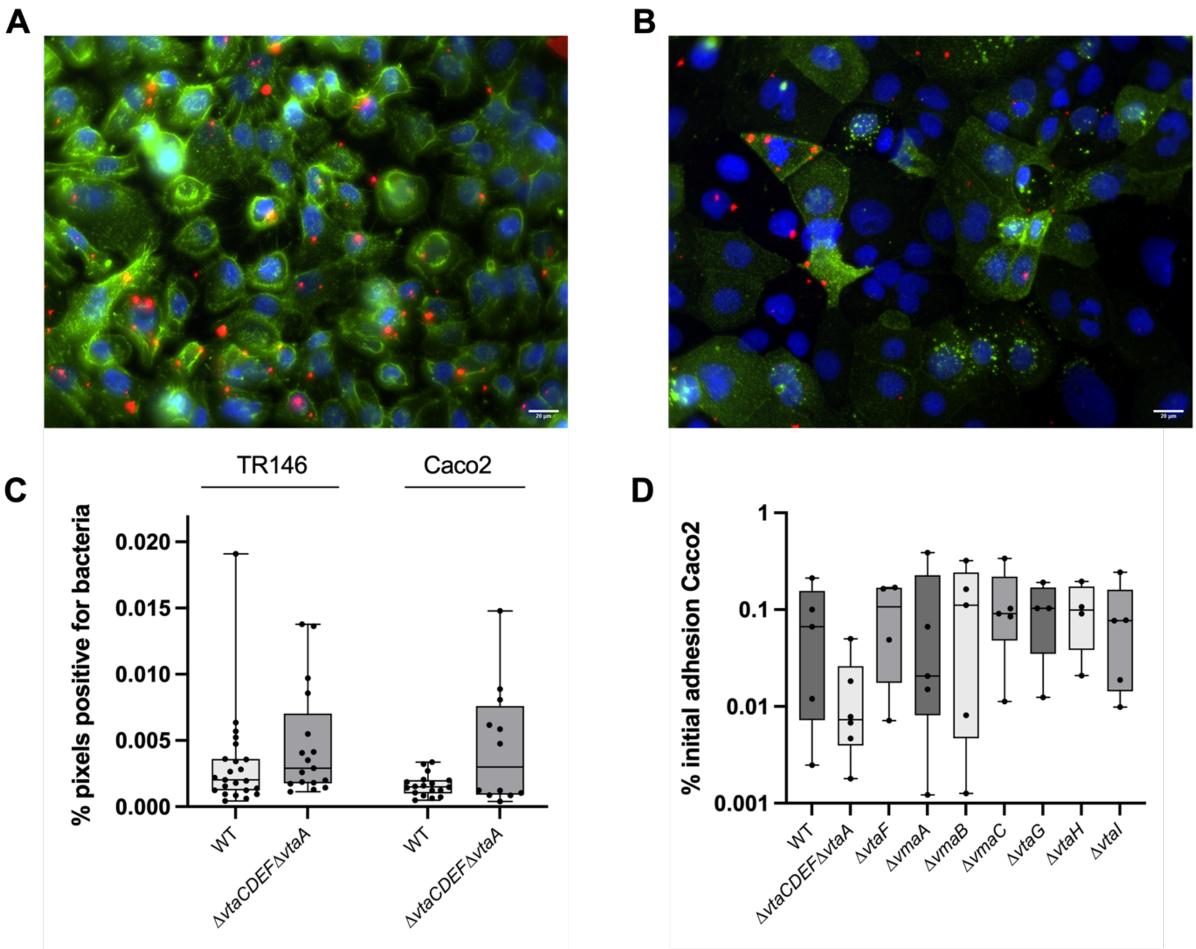

Supplementary Figure S7: *V. parvula* presents a limited adhesion to host cells. Representative image of *V. parvula* SKV38 WT adhesion on buccal TR146 (A) and intestinal Caco-2 epithelial cells (B) after 30min. Bacteria are labelled in red, human cell surface labelled in green and nuclei in blue. Scale bar is 20 µm. (C) Quantification of the adhesion shown in A-B, (N=2) as the percentage of pixels positive for bacteria in the Z-stack (See supplementary methods). No statistical differences were observed between the different groups using a Kruskal-Wallis test. (D) Ratio of adhering bacteria compared to the total amount of bacteria after 2h of incubation on Caco-2 cells for *V. parvula* WT and different adhesin mutants (N=4 to 5).

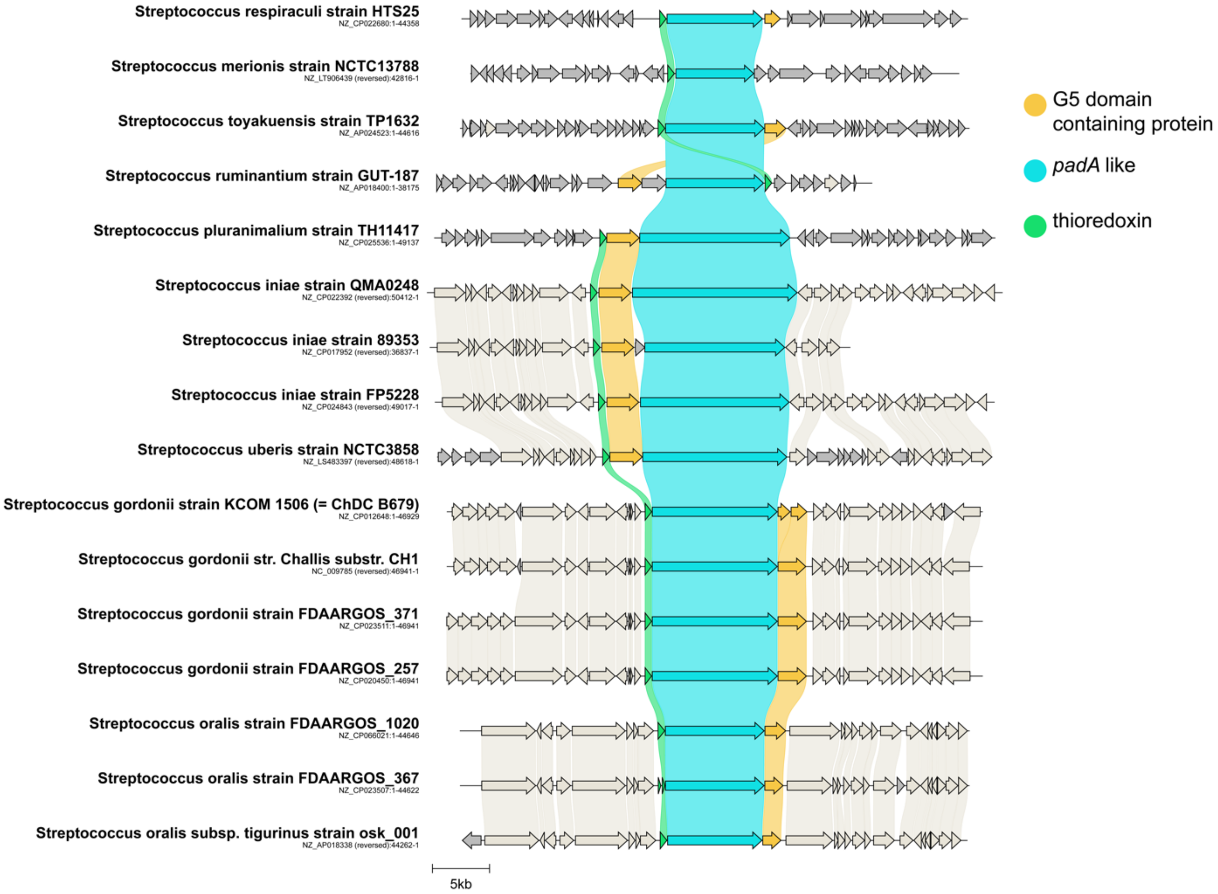

Supplementary Figure S8: Conservation of the *padA* operon in distant streptococci.

A local subset of Streptococcus genomes was searched for presence of PadA using a custom HMM profile. Neighboring regions were extracted and aligned using clinker(Gilchrist and Chooi 2021). *padA* homologs are colored in blue, neighboring G5 domain containing proteins in yellow and thioredoxins in green.

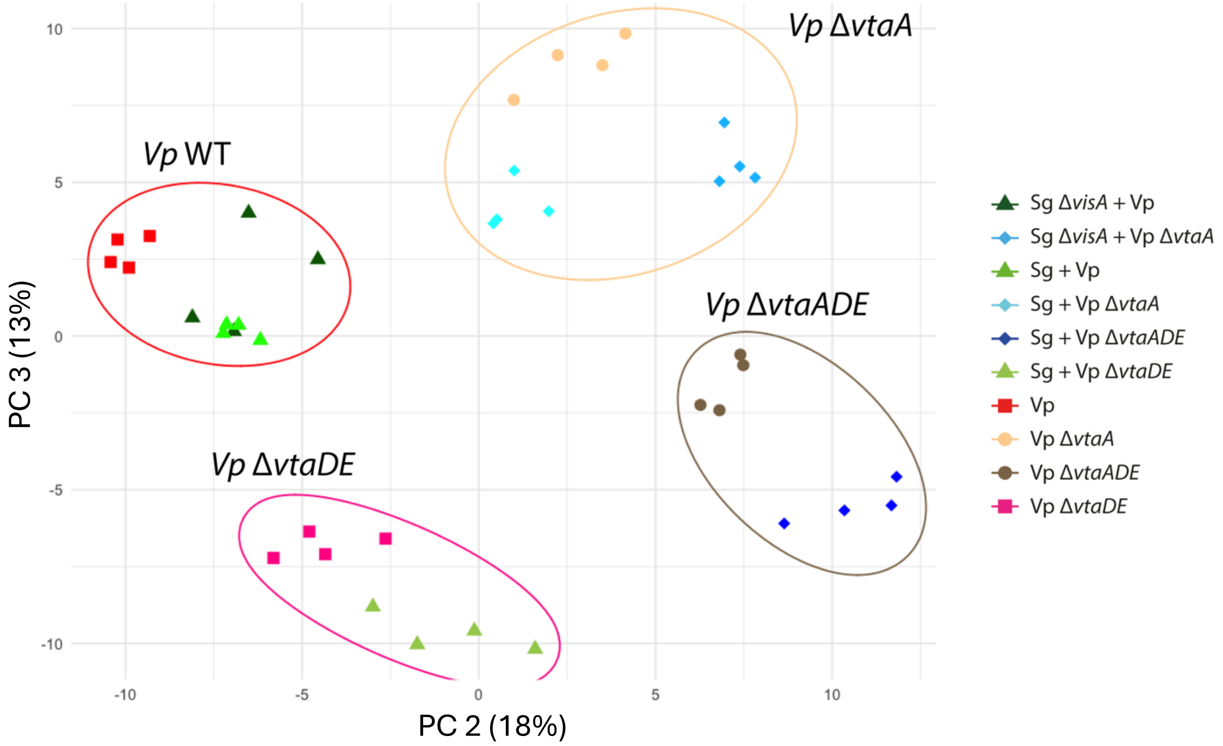

Supplementary Figure S9: 2nd and 3rd dimension of principal component analysis (PCA) of all *V. parvula* samples.

These experiments correspond to 4 biological replicates for 10 conditions. Colors and shape represent the different conditions. The four circles separate samples based on *Veillonella* strains.

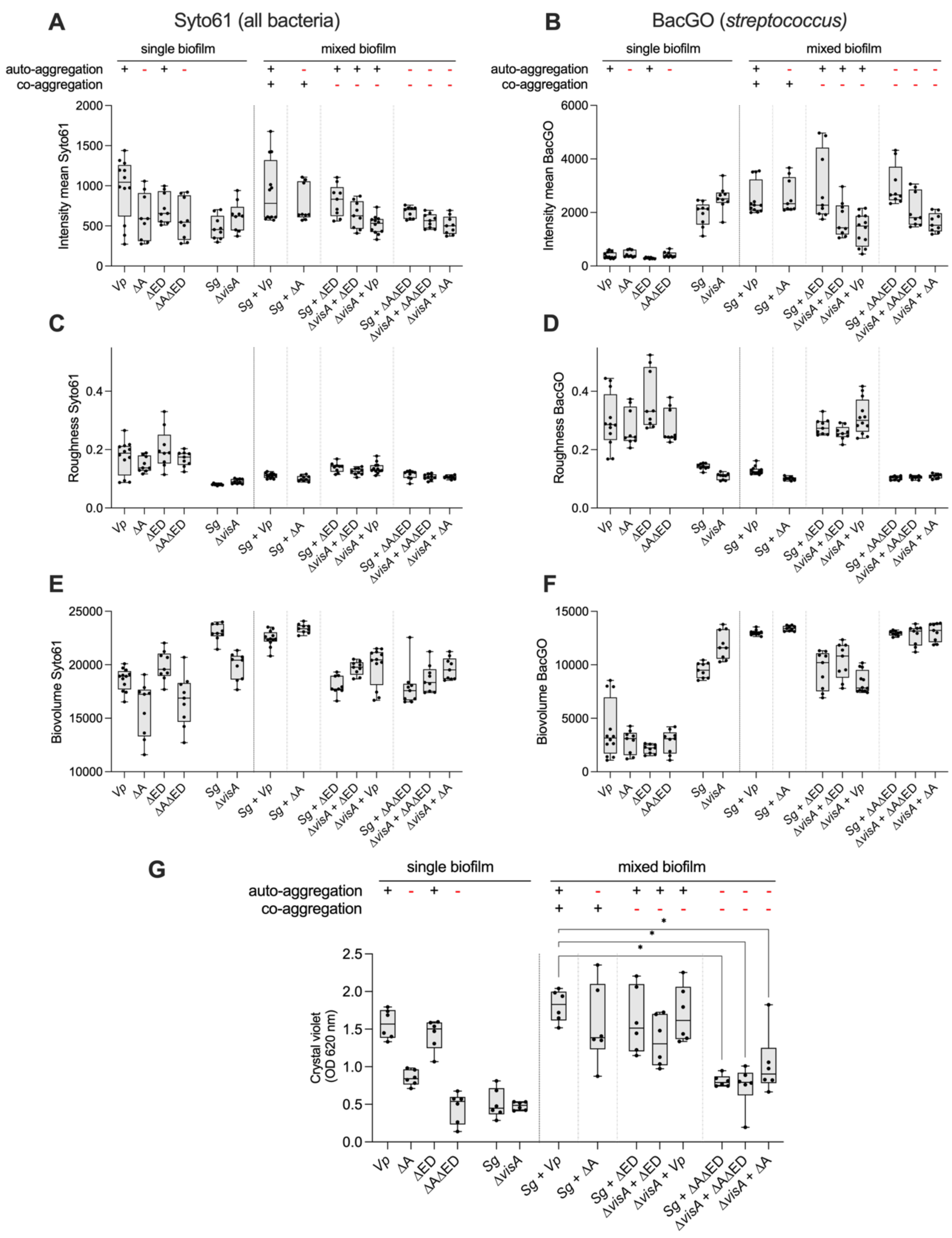

Supplementary Figure S10: **Biofilms calculated parameters**.

Average intensity of each channel (A-B), biofilm roughness (C-D) and the Biofilm volume (E-F) were calculated for the BacGO dye (*Streptococcus*) and Syto61 dye (all bacteria) using BiofilmQ 1.1. Each point (9-12 per condition) represents the average roughness measurement of four images per well. Experiment done in three biological independent replicates. G) Estimations of biofilm formation in BHIP medium in a 96-well plate by crystal violet staining. Min-max box plots for 6 biological replicates. Each replicate is the mean of two technical replicates. A Kruskal-wallis test comparing all mixed biofilms conditions to *Sg -V. parvula* followed by a Benjamini, Krieger and Yekutieli correction for multiple testing was used. Significant P values (<0.05) are indicated by an asterisk. For all plots, *Vp* is *V. parvula*, ΔA is *V. parvula ΔvtaA*, ΔED is *V. parvula ΔvtaD-vtaE*, *Sg* is *S. gordonii* and Δ*visA* is *S. gordonii ΔvisA*. Presence (or absence) of auto- and co- aggregation is indicated by the + (or -) symbols.

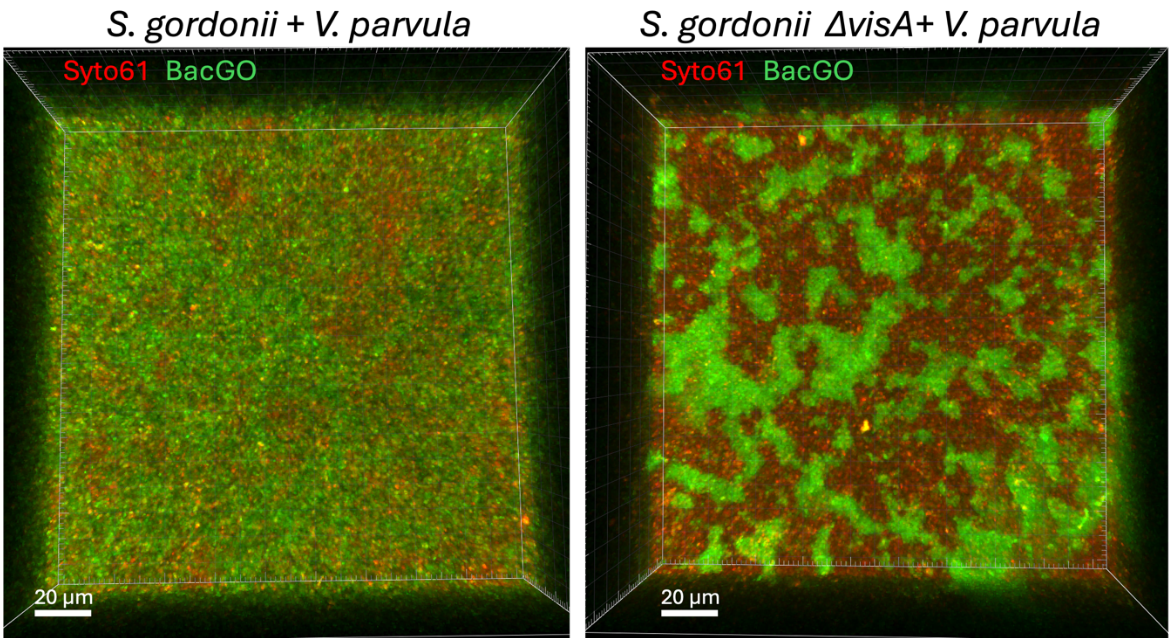

Supplementary Figure S11: **Additional microscopy images of mixed biofilms**. Three-dimensional reconstruction of the biofilm from CLSM data (scale bar is 20 μm). For better visualization of the mixed biofilms, these were imaged using a Leica TCS SP8 confocal fluorescent microscope (INRAE MIMA2 microscopy platform) with a 63X water immersion lens. The initial adhesion was done at OD600 1 instead of the 0.05 used for the quantification. Scale bar is 20 μm. Images were processed using Imaris 9.0. The green signal corresponds to the BacGO dye (*Streptococcus gordonii*) and the red signal to the Syto61 dye (all bacteria).

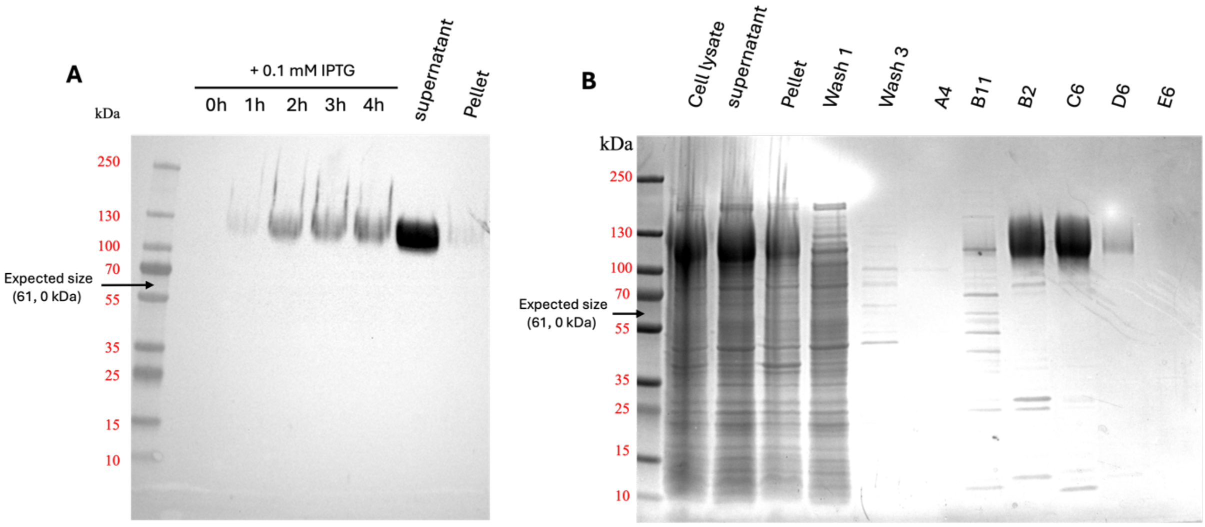

Supplementary Figure S12: Purification of $VisA_{G5}$

A) Anti His-Tag Western blot on *E. coli* whole cell lysates at different times after protein production induction by IPTG. Supernatant and pellet correspond to the different fractions of a 4h induced *E. coli* lysate by BugBuster Protein Extraction Reagent. B) Whole protein gel stained with SimplyBlue SafeStain corresponding to the different steps of protein purifications. 60 fractions (A1 to E12) were eluted and collected among which selected fractions were resuspended in Laemmli buffer with 2.5% β-mercaptoethanol to break potential disulfide bonds and deposited on the presented gel. Fractions B6 to D10 were pooled together to yield the final purified protein. The arrows indicate the expected size of a monomer of the purified protein. The VisAG5 protein is apparently capable of forming multimers, at least dimers, that are resistant to the denaturing conditions of SDS PAGE analysis.

Supplementary methods

Biofilm formation in 96-well microtiter plates.

Overnight cultures of *S. gordonii* strains in BHI and *V. parvula strains* in BHIL medium were diluted to an OD_{600} of 0.05 and transferred to two flat-bottom TPP 96-well plates (Dustscher), adding 150 µL per well. After 24 h of static incubation in anaerobia, supernatant was removed, and one of the two plates was resuspended by pipetting to measure OD_{600} using a Tecan Infinite-M200-Pro spectrophotometer. The other plate was used for coloration. Biofilms were fixed with 150 µL Bouin solution (HT10132; Sigma-Aldrich) for 15 min. Bouin solution was removed by inversion, and the biofilms were washed once in water. The biofilms were stained with 150 µL of 1% crystal violet (V5265; Sigma-Aldrich) for 15 min without shaking and then washed in water three times and left to dry. All washes were made by flicking the plate. After drying the plate, crystal violet was dissolved with 200 µL absolute ethanol and OD_{620} was measured.

Culture of the human epithelial cells

Caco-2 and TR146 (CancerTools Ref 151425) cells were maintained in DMEM Glutamax (Gibco) supplemented with 10% of heat-inactivated serum and non-essential amino acids (Gibco). Caco2 cells were grown at 37°C in a 10% CO2 incubator and TR146 cells in a 5% CO2 incubator. Contamination with mycoplasma was regularly tested using PCR test and HEK hTLR2 (Invivogen).

Adhesion of *V. parvula* adhesin mutants on Caco-2 cells

The day prior each experiment, fresh cultures of *V. parvula* strains were inoculated and Caco-2 cells were seeded at 0.4 x 10^6 cells per well in 12 wells plates (TPP). On the day of experiment, Caco-2 medium was removed and changed to DMEM without serum and placed in the anaerobic chamber (Jacomex) at least 1h before the adhesion. In the anaerobic chamber, *V. parvula* strains were centrifuged at 5000g for 10min at room temperature, washed with PBS, centrifuged again before resuspension in DMEM without serum at OD=1. For the adhesion, 10 µL of each strain/well were added and incubated for 2h at 37°C. After incubation, the supernatant was removed from each well, and Caco-2 cells were washed 3x with 1 mL PBS before adding 50µL of Trypsin-EDTA 0.25% (Gibco) and 15min incubation at 37°C. To resuspend Caco-2 cell lysate, 50 µL of PBS was added and homogenised by pipetting. Serial dilutions for

each mutant were performed from 10^{-1} to 10^{-7} in PBS and plated in duplicate on SK agar plates and left for incubation at 37°C for 2 days.

Comparison of adhesion on buccal and intestinal epithelial cells by microscopy

Two days before the experiment, Caco-2 and TR146 cells were seeded at 0.4x 10^6 cells per well on glass cover slips in 24 wells plate. One day prior experiment the bacterial cultures were refreshed. On the day of experiment, 1 mL of culture of each strain were harvested by centrifugation at 5000g for 8min. Bacteria were resuspended in PBS at OD=1 and 0.5µL of Cellbrite 640 (Biotium) was added to 500 µl of bacterial and incubate 30min at 37°C with shaking at 300rpm. To wash the dye, 1 mL of PBS was added before centrifugation at 5000 g for 8 min and bacteria were resuspended in PBS. To dislodge aggregates, bacteria were sonicated 2 x 10s in a water bath sonicator and 100 µM of each bacterial culture was added to each epithelial cell well and let to adhere for 30min at 37°C. After the incubation, the wells were washed 3 times with PBS before fixation with PFA 4% for 40min at room temperature. After fixation and, cells were washed with PBS and cell surface was stained with WGA-555 10 µg/mL (Invivogen) for 45min, washed with PBS and stained with DAPI 2 µg/mL for another 10 min, all these steps were done at room temperature. After 2 final washes with PBS, cover slips were mounted with Prolong Gold and cured overnight.

A minimum of 5 fields were taken with Z stack for each cover slip, with 40X objective on a IX81 Olympus microscope. Each image was chosen on the DAPI channel and acquired on the 3 channels (DAPI, Cy3, Cy5). Images were quantified with Fiji by duplicating each image on the Cy5 channel and applying an automated threshold (Otsu method). Pixels occupied by the bacteria for each image of the stack were summed as well as the total number of pixels and ratios were calculated for each field.

Supplementary references

Ajdić, Dragana, William M. McShan, Robert E. McLaughlin, Gorana Savić, Jin Chang, Matthew B. Carson, Charles Primeaux, et al. 2002. "Genome Sequence of Streptococcus Mutans UA159, a Cariogenic Dental Pathogen." *Proceedings of the National Academy of Sciences* 99 (22): 14434–39. https://doi.org/10.1073/pnas.172501299.

Béchon, Nathalie, Alicia Jiménez-Fernández, Jerzy Witwinowski, Emilie Bierque, Najwa Taib, Thomas Cokelaer, Laurence Ma, Jean-Marc Ghigo, Simonetta Gribaldo, and Christophe Beloin. 2020. "Autotransporters Drive Biofilm Formation and Autoaggregation in the Diderm Firmicute Veillonella Parvula." *Journal of Bacteriology* 202 (21):e00461-20. https://doi.org/10.1128/JB.00461-20.

Evans, Richard, Michael O'Neill, Alexander Pritzel, Natasha Antropova, Andrew Senior, Tim Green, Augustin Žídek, et al. 2022. "Protein Complex Prediction with AlphaFold-Multimer." bioRxiv. https://doi.org/10.1101/2021.10.04.463034.

Gilchrist, Cameron L M, and Yit-Heng Chooi. 2021. "Clinker & Clustermap.Js: Automatic Generation of Gene Cluster Comparison Figures." *Bioinformatics* 37 (16): 2473–75. https://doi.org/10.1093/bioinformatics/btab007.

Knapp, Steven, Clint Brodal, John Peterson, Fengxia Qi, Jens Kreth, and Justin Merritt. 2017. "Natural Competence Is Common among Clinical Isolates of Veillonella Parvula and Is Useful for Genetic Manipulation of This Key Member of the Oral Microbiome." *Frontiers in Cellular and Infection Microbiology* 7:139 https://doi.org/10.3389/fcimb.2017.00139.

Lee, S F, A Progulske-Fox, G W Erdos, D A Piacentini, G Y Ayakawa, P J Crowley, and A S Bleiweis. 1989. "Construction and Characterization of Isogenic Mutants of Streptococcus Mutans Deficient in Major Surface Protein Antigen P1 (I/II)." *Infection and Immunity* 57 (11): 3306–13. https://doi.org/10.1128/iai.57.11.3306-3313.1989.

Madeira, Fábio, Matt Pearce, Adrian R N Tivey, Prasad Basutkar, Joon Lee, Ossama Edbali, Nandana Madhusoodanan, Anton Kolesnikov, and Rodrigo Lopez. 2022. "Search and Sequence Analysis Tools Services from EMBL-EBI in 2022." *Nucleic Acids Research* 50 (W1): W276–79. https://doi.org/10.1093/nar/gkac240.

Nairn, Brittany L., Grace T. Lee, Ashwani K. Chumber, Patrick R. Steck, Mahmoud O. Mire, Bruno P. Lima, and Mark C. Herzberg. 2020. "Uncovering Roles of Streptococcus Gordonii SrtA-Processed Proteins in the Biofilm Lifestyle." *Journal of Bacteriology* 203 (2):e00544-20. https://doi.org/10.1128/JB.00544-20.

Pettersen, Eric F., Thomas D. Goddard, Conrad C. Huang, Elaine C. Meng, Gregory S. Couch, Tristan I. Croll, John H. Morris, and Thomas E. Ferrin. 2021. "UCSF ChimeraX: Structure Visualization for Researchers, Educators, and Developers." *Protein Science: A Publication of the Protein Society* 30 (1): 70–82. https://doi.org/10.1002/pro.3943.

Switalski, L. M., and W. G. Butcher. 1994. "An in Vitro Model for Adhesion of Bacteria to Human Tooth Root Surfaces." *Archives of Oral Biology* 39 (2): 155–61. https://doi.org/10.1016/0003-9969(94)90111-2.
